# Diagnostic dilemma in a 3-year-old girl with acute nephritic syndrome and hematologic abnormalities: Questions

**DOI:** 10.1007/s00467-022-05733-9

**Published:** 2022-10-17

**Authors:** Samantha Innocenti, Silvia Bernardi, Maud Prévot, Antonin Saldmann, Maud Tusseau, Alexandre Belot, Jean-Paul Duong Van Huyen, Olivia Boyer

**Affiliations:** 1 Néphrologie Pédiatrique, Centre de Référence MARHEA, Hôpital Necker-Enfants Malades, APHP, Institut Imagine, Inserm U1163, Université Paris Cité, Paris, France; 2 Nephrology and Dialysis Unit, Meyer Children’s Hospital, Florence, Italy; 3 School of Nephrology, Università Degli Studi Di Milano, ASST Papa Giovanni XXIII, Bergamo, Italy; 4 Immunology Department, Hopital Européen Georges Pompidou, APHP, Paris Cité University, Paris, France; 5 Centre International de Recherche en Infectiologie, Univ Lyon, Inserm, U1111, Université Claude Bernard, Lyon 1, Centre National de La Recherche Scientifique, UMR5308, ENS de Lyon, Lyon, France; 6 Pediatric Nephrology, Rheumatology, Dermatology Department, Hôpital Femme Mère Enfant, CRMR RAISE, Hospices Civils de Lyon, Bron, France; 7 The International Center of Research in Infectiology, Lyon University, INSERM U1111, CNRS UMR 5308, ENS, UCBL, Lyon, France; 8 Department of Pathology, Necker-Enfants Malades Hospital, APHP, Paris Cité University, Paris, France

**Keywords:** Macrohematuria, Proteinuria, Hypocomplementemia, Anemia, Thrombocytopenia, Childhood

## Case

A 3-year-old girl presented to the Emergency Department with the chief complaint of brown macrohematuria for 2 weeks. Her past medical history revealed scarlet fever and mild upper respiratory tract infections 2 months earlier, and no recent trauma or travel was reported. She had no familiarity for rheumatological disease, and her parents were unrelated. The first clinical examination was unremarkable, including the absence of fever, arthralgia, or skin rash. The patient presented with normal growth and development and up-to-date vaccinations. Abdominal ultrasound reported bilateral kidney hyperechogenicity with a preserved cortico-medullary differentiation, and chest radiography was normal. Initial blood and urinary tests are summarized in Table 1 and mainly revealed a non-hemolytic anemia associated with thrombocytopenia, positive direct Coombs test, hypergammaglobulinemia, and high ESR:CRP ratio. Kidney function was normal, and urinalysis revealed hematuria associated with proteinuria. Furthermore, a persistent activation of the classical complement pathway was noticed in association with a low CH50 and the presence of anti-C1q and anti-C3b antibodies.

**Table 1:** Laboratory tests at referral

	*At referral*	*Normal values*
Hemoglobin (g/dL)	8.8	11.5–14
Leukocytes (× 10 ^9^ /L)	6.44	5.5–15
Platelets (× 10 ^9^ /L)	62	150–450
Reticulocytes (× 10 ^9^ /L)	141.0	20–100
Haptoglobin (g/L)	3.4	0.3–2
Total bilirubin (μmol/L) ESR (mm)	3 71	0.8–6.8 < 10
Schistocytes (%)	< 1	< 1
LDH (UI/L)	322	192–321
Albumin (g/L)	13.2	34–50
Proteins (g/L)	57	64–82
Ferritin (mcg/L)	449	12–224
Triglycerides (mmol/L)	3.94	0.5–2,23
BUN (mmol/L) Creatinine (μmol/L) eGFR (beside Schwartz formula, mL/min/1.73 m^2^)	4.8 29 101	2.5– 6.4 15–34 > 90
Proteinuria (g/L)	2.87	0–0.1
Urine creatinine (mmol/L)	1.8	1–5
Urinary P/C ratio (g/mmol)	1.6	
D-dimer (ng/mL)	79,131	< 500
Activated fibrinogen (g/L)	1	2–4
aPTT (s)	45	25–38
Prothrombin ratio (%) INR	76 1.14	70–150 0.9–1.2
Antithrombin III (%) Direct Coombs test	75 Positive	80–120 Negative
C3 (mg/L)	239	660–1250
C4 (mg/L)	68	93–380
CH50 (%)	19	70–130
sC5b9 (ng/mL) Anti-C1q (UA) Anti C3b antibodies IgG Anti-CFH antibodies Anti-CFB antibodies	> 1820 204 Positive (598 UA) Negative Negative	< 300 < 30 Negative Negative Negative
ANA	> 1:800	< 1:80
Anti-dsDNA antibodies (U/mL)	163	Negative
ENA	Anti-Sm + , anti-RNP + , antiSSA (Ro) +	Negative
Anti-platelet antibodies	Positive	Negative
Anti-beta2GP1 antibodies IgG (U/mL) Anti-cardiolipin antibodies IgG	64 Negative	< 20 Negative
Lupus anticoagulant Anti-phosphatidylserine/thrombin antibodies IgG (U) Anti-phosphatidylserine/thrombin antibodies IgM (U)	Positive > 150 > 150	Negative < 30 < 30

After 3 days, the patient presented malar rash, palatal petechiae, and peripheral edema associated with a rapid worsening of anemia and thrombocytopenia.

Autoimmune screen revealed high titer of ANA, anti-dsDNA, ENA, and p-ANCA with anti-platelet, anti-beta2GP1, and anti-phosphatidylserine antibodies associated with LAC positivity but no anti-complement factor B (CFB) and anti-complement factor H (CFH) autoantibodies.

## Questions

1. Which major diagnoses must be considered in this context?
2. What further investigations would you perform for the work-up of your main hypothesis?
3. If the main hypothesis is confirmed, which treatment regimen could you consider?

